# Evaluation and Optimization of Pharmaceutical Inventory Management in a Tertiary Care Teaching Hospital in Jharkhand, India, Using ABC-VED Analysis

**DOI:** 10.7759/cureus.99406

**Published:** 2025-12-16

**Authors:** Sahil Sinha, Bhoopendra Singh

**Affiliations:** 1 Hospital Administration, Rajendra Institute of Medical Sciences, Ranchi, IND; 2 Forensic Medicine and Toxicology, Rajendra Institute of Medical Sciences, Ranchi, IND

**Keywords:** abc–ved analysis, drug management, hospital pharmacy, pharmaceutical inventory, resource optimization, tertiary care hospital jharkhand

## Abstract

Background

Efficient pharmaceutical inventory management is crucial for ensuring uninterrupted patient care, especially in resource-limited tertiary hospitals. Inappropriate stock control can lead to both financial inefficiencies and drug shortages, affecting clinical outcomes. This study aimed to evaluate the pharmaceutical inventory practices of a tertiary care teaching hospital in Jharkhand, India, using ABC (Always, Better, Control), VED (Vital, Essential, Desirable), and integrated ABC-VED analyses to identify expenditure patterns and critical control priorities.

Methods

A descriptive cross-sectional study was conducted at the central medical store of a tertiary care teaching hospital. Ethical approval was obtained from the Institutional Ethics Committee, vide letter no. 161 dated 05.03.2025. Data pertaining to all pharmaceutical items procured, stored, and dispensed between April 2022 and March 2023 were extracted from the hospital’s electronic inventory and verified with physical ledgers. Non-drug items were excluded. Each item’s annual consumption value (quantity × unit price) was used for ABC classification, while clinical criticality determined VED categorization. The integrated ABC-VED matrix was then developed to identify high-priority drugs requiring stringent control. Data were analyzed descriptively using Microsoft Excel for Mac, Version 16.103.4 (Microsoft Corp., Redmond, WA, USA).

Results

A total of 192 stock-keeping units (SKUs) worth ₹106.75 million were analyzed. ABC analysis revealed that only 2.6% of items (Category A) accounted for 39.97% of total expenditure, whereas 10.4% (Category B) and 86.9% (Category C) contributed 39.64% and 20.39% respectively. VED analysis showed that Vital items comprised 8.3% of SKUs (6.05% of cost), Essential 18.2% (13.82%), and Desirable 73.4% (80.13%). Integration of both methods identified that a small subset of high-cost, high-priority drugs (Category AV) requires continuous monitoring, while a substantial portion of expenditure is directed toward costly but low-criticality drugs (AD and BD). Operational reviews highlighted deficiencies in stock tracking and reactive procurement practices, contributing to mismatched stock levels.

Conclusion

The ABC-VED matrix proved to be a valuable tool for optimizing inventory control by linking financial significance with clinical importance. Strengthening digital stock management, enforcing strict monitoring of Category I (high-value, Vital) items, and adopting proactive procurement strategies can enhance efficiency and ensure uninterrupted drug availability in tertiary care hospitals.

## Introduction

Pharmaceutical inventory management is critical for the effective operation of hospitals and the delivery of patient care. According to the World Health Organization (2019), efficient health resource utilization is central to maintaining care quality and ensuring patient safety [1]. In tertiary teaching hospitals, where patient volume is high and care is multidisciplinary, maintaining the availability of medicines, consumables, and equipment is essential to avoid treatment interruptions and prevent adverse outcomes. Hospital inventories extend beyond pharmaceuticals to include surgical tools, laboratory reagents, and protective equipment-each vital for daily healthcare functions [2].

However, many hospitals in India, particularly in resource-limited regions such as Jharkhand, India, still rely on manual or semi-manual inventory systems such as spreadsheets and paper records. As Devnani et al. (2010) noted, these traditional methods are often inefficient, prone to human error, and lack real-time visibility, which is an issue that becomes critical during emergencies or sudden surges in demand [3]. Consequently, hospitals frequently experience stockouts, overstocking, and drug wastage, which compromise both patient care and financial efficiency [4-5]. These challenges are especially pronounced in public-sector facilities, where procurement delays and fluctuating demand further increase the risk of essential medicine shortages [6].

Recent global trends emphasize the need for digital transformation in hospital supply chains. Technologies such as barcoding, radio-frequency identification (RFID), and cloud-based inventory systems have been shown to improve monitoring accuracy, enable automated replenishment, and integrate with electronic health records [7]. For example, Mulac et al. (2021) and Wu Yi Zheng et al. (2021) demonstrated that barcode medication administration (BCMA) systems substantially reduce dispensing errors and strengthen control of high-value medicines [8,9]. Similarly, RFID-based platforms allow real-time tracking of medical supplies and pharmaceuticals, improving compliance and operational efficiency (Lee & Lee, 2011; Álvarez López et al., 2018) [10,11].

To further enhance efficiency, analytical tools such as ABC (Always, Better, Control) and VED (Vital, Essential, Desirable) classifications are widely used in hospital inventory control [3,4]. The ABC technique ranks items by their annual consumption value-typically categorizing the top 70% of expenditures as class A, the next 20% as class B, and the remaining 10% as class C (Devnani et al., 2010; Gupta et al., 2007) [3,4]. In contrast, the VED analysis prioritizes items based on their criticality to patient care, classifying them as vital, essential, or desirable (Vaz et al., 2008) [2]. When combined, the ABC-VED matrix provides a structured framework to identify both cost-intensive and clinically critical items, enabling better financial control and uninterrupted drug availability (Jobira et al., 2021; Yohannes et al., 2022) [5,6].

Given these considerations, this study evaluates pharmaceutical inventory management in a resource-constrained tertiary-care teaching hospital in Jharkhand using ABC and VED analyses. The objective is to identify operational inefficiencies and propose strategies to optimize resource allocation, reduce wastage, and ensure consistent drug availability. Ultimately, this approach supports the development of resilient, evidence-based inventory systems that enhance hospital performance and sustainability in resource-limited environments.

## Materials and methods

Study design and setting

This descriptive cross-sectional study was conducted in the Central Medical Store of a tertiary care teaching hospital located in Jharkhand, India. The hospital provides comprehensive medical, surgical, and emergency services to a large and diverse patient population, including rural and economically underprivileged communities. The setting was selected to represent the operational realities of a public-sector hospital in a resource-constrained environment, where efficient pharmaceutical inventory management is essential to ensure uninterrupted patient care and optimal utilization of limited financial resources.

Study duration and data source

The study analyzed pharmaceutical inventory data for the financial year April 2022 to March 2023. Data collection and verification were conducted during March-April 2025. Information was obtained from the hospital’s physical inventory registers, procurement ledgers, and stock records maintained at the Central Medical Store.

Only pharmaceutical items procured, stored, and dispensed through the hospital’s central store were included in this analysis, whereas surgical items, medical devices, and other non-drug consumables were excluded. For each medicine, information on its generic name, formulation, quantity procured, unit cost, applicable Goods and Services Tax (GST) rate, total annual expenditure, and cumulative contribution to overall drug spending was collected. All procurement data were cross-verified with supplier invoices and ledger entries to ensure completeness and accuracy.

Inventory classification methods

ABC Analysis

The ABC (Always, Better, Control) method was applied by calculating the annual consumption value for each item as the product of quantity procured and unit cost. Items were arranged in descending order of expenditure, and cumulative cost percentages were plotted to derive category cutoffs empirically from the expenditure distribution rather than using predetermined 70/20/10 thresholds. Category A included the small subset of items accounting for approximately 70% of total expenditure, Category B represented items contributing the next 20%, and Category C comprised the large number of low-cost items contributing the remaining 10%. This approach allowed identification of high-expenditure medicines requiring tighter managerial oversight.

VED Classification

Drugs were simultaneously classified using the VED (Vital, Essential, Desirable) system based on clinical importance. Vital drugs were defined as life-saving or emergency-use items requiring uninterrupted availability, Essential drugs were those routinely used in standard therapeutic protocols, and Desirable drugs were supportive items that could tolerate temporary stockouts without compromising patient care. Classification was conducted in consultation with senior clinicians and pharmacists serving on the Drug and Therapeutics Committee to ensure clinical relevance.

ABC-VED Matrix

An integrated ABC-VED matrix was then constructed by cross-tabulating cost-based and criticality-based categories, resulting in nine possible combinations. These combinations were consolidated into three operational groups: Category I (AV, AE, AD, BV), representing high-priority items requiring stringent control and zero-tolerance stockout; Category II (BE, BD, CE), representing items needing periodic review and cost rationalization; and Category III (CD), representing low-cost and low-criticality medicines manageable through flexible procurement. This combined analytical approach enabled a balanced assessment of both financial impact and clinical necessity, supporting rational procurement and efficient resource allocation.

Data Analysis

The annual consumption value for each drug was calculated as the product of the quantity procured and the unit cost. All data were compiled, cleaned, and analyzed using Microsoft Excel for Mac, Version 16.103.4 (Microsoft Corp., Redmond, WA, USA). Descriptive statistics, including frequencies, percentages, and cumulative cost shares, were generated to summarize expenditure patterns and category-wise distribution across ABC, VED, and ABC-VED classifications. Tables and graphical representations were used to illustrate the distribution of medicines within each category.

To statistically assess whether the observed ABC, VED, and ABC-VED category distributions deviated significantly from expected patterns, a Chi-square goodness-of-fit test was applied. Expected frequencies were derived from conventional benchmark proportions commonly reported in earlier hospital-based inventory studies. The Chi-square test provided an objective measure of the extent to which the hospital’s inventory distribution aligned with or differed from standard inventory norms. A p-value <0.05 was considered statistically significant. This combined analytical approach enabled both descriptive and inferential evaluation of the drug inventory profile, helping identify categories of high-cost or clinically critical items that require closer managerial control.

Ethical approval

The study protocol was reviewed and approved by the Institutional Ethics Committee (IEC) vide letter no. 161 dated 05.03.2025, prior to initiation. As the analysis utilized secondary data from existing store records without any patient identifiers, the requirement for individual informed consent was waived.

## Results

A total of 192 distinct stock-keeping units (SKUs) were included in the analysis, representing the pharmaceutical inventory of the tertiary care hospital for the financial year 2022-2023. The total annual drug expenditure was ₹106.75 million. Inventory data were analyzed using ABC, VED, and integrated ABC-VED matrix methods.

ABC classification

The ABC analysis revealed a distinct cost concentration pattern (Table 1). Only 5 (2.6%) of SKUs (Category A) accounted for nearly 42.67 (39.64%) of total expenditure, emphasizing that a small number of high-cost medicines dominate financial consumption. Category B, representing 20 (10.4%) of SKUs, contributed (42.32) 39.64% of costs, while the majority of items (Category C, 167 (86.9 %) accounted for only 21.76 (20.39%) of total expenditure. This pattern reflects a classic Pareto distribution, where targeted control of a small subset of items can significantly impact cost efficiency (Table 1).

**Table 1:** ABC analysis of inventory items A: Always; B: Better; C: Control; SKUs = stock-keeping units; ₹M = million Indian rupees Percentages are rounded to two decimals. The total cost represents cumulative annual drug expenditure (₹106.75 million).

ABC category	SKUs n (%)	Total cost (₹M) n (%)	Average cost per SKU (₹M)
Always (A)	5 (2.6%)	42.67 (39.97%)	8.53
Better (B)	20 (10.4%)	42.32 (39.64%)	2.12
Control (C)	167 (86.9%)	21.76 (20.39%)	0.13
Total	192	106.75	—

ABC Cumulative Expenditure Curve

**Figure 1:**
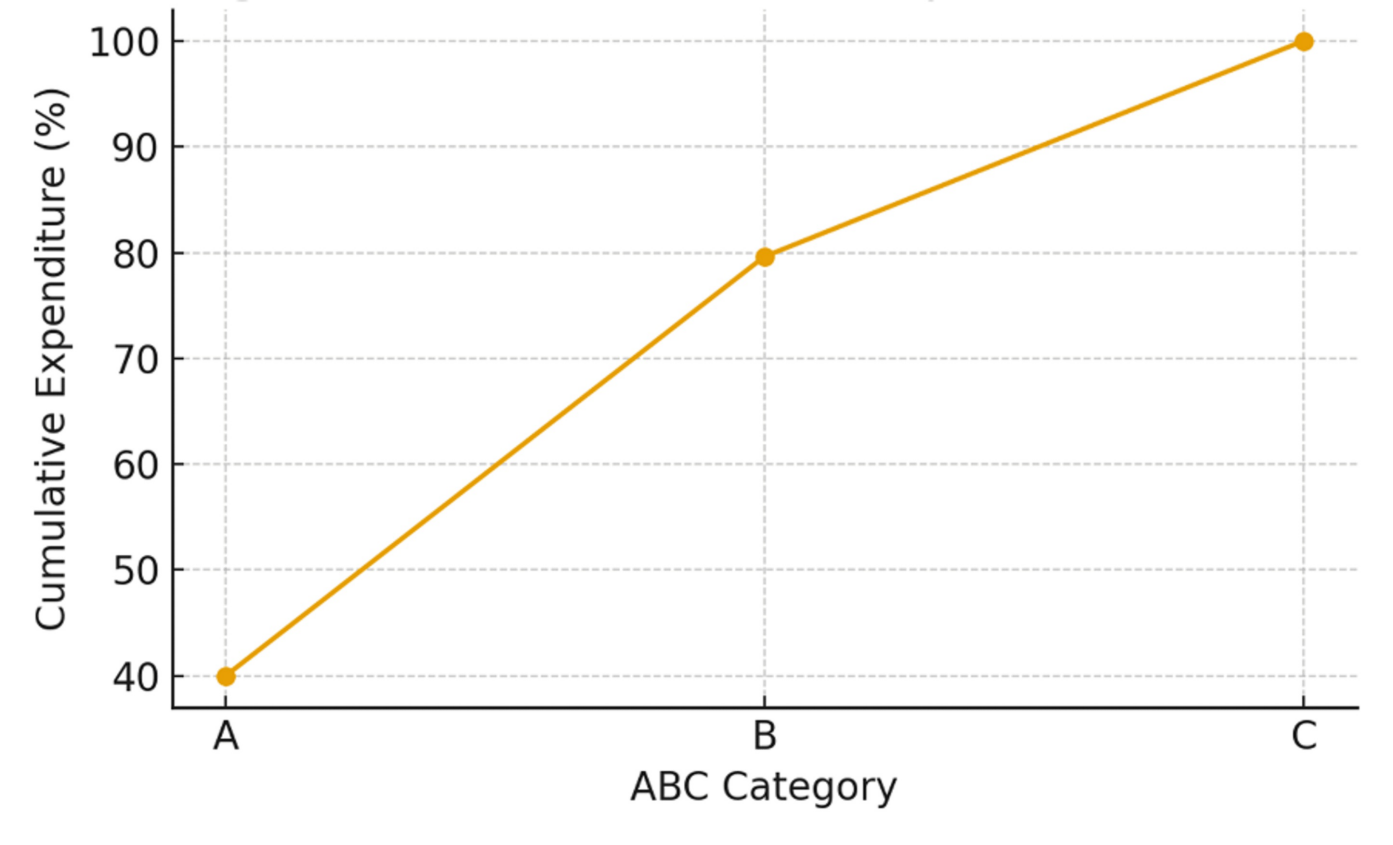
ABC cumulative expenditure curve A: Always; B: Better; C: Control; SKUs = stock-keeping units

Cumulative expenditure curve depicting the proportional cost contribution of inventory items classified under ABC analysis. Category A items, representing only five (2.6%) of total stock-keeping units (SKUs), accounted for nearly 40% of total expenditure, highlighting concentrated spending on a few high-value items. Category B covered 20 (10.4%) of SKUs with 39.6% of expenditure, while Category C comprised the majority, 167 (86.9%), but only 20.4% of total cost (Figure 1). 

Statistical Interpretation

A Chi-square goodness-of-fit test was applied to compare the distribution of SKUs with the distribution of total expenditure across A, B, and C categories. The test demonstrated a highly significant deviation between SKU proportions and cost proportions (p < 0.001), indicating that pharmaceutical expenditure is strongly concentrated in a very small number of items. Although category A comprised only 2.6% of total SKUs, it accounted for 39.97% of total expenditure, whereas category C represented 86.9% of SKUs but only 20.39% of expenditure. This statistically significant imbalance supports the relevance of ABC analysis for prioritizing managerial oversight of high-cost, low-frequency items.

VED classification

The VED classification assessed the clinical importance of each drug (Table 2).

**Table 2:** VED classification of inventory items V = Vital; E: Essential; D: Desirable;  SKUs = stock-keeping units; ₹M = million Indian rupees Percentages are rounded to two decimals. ‘Average cost/SKU’ indicates mean expenditure per item within each category.

VED category	SKUs n (%)	Total cost (₹M) n (%)	Average cost/SKU (₹M)
Vital (V)	16 (8.3%)	6.46 (6.05%)	0.40
Essential (E)	35 (16.2%)	14.75 ( 13.82%)	0.42
Desirable (D)	141 (73.4%)	85.54 (80.13%)	0.61
Total	192 (100%)	106.75 (100%)	—

Vital drugs comprised 16 (8.3%) of SKUs, accounting for 6.46 (6.05%) of expenditure. Essential drugs made up 35 (18.2%) of SKUs and 14.75 (13.82%) of total costs. Desirable drugs dominated the inventory in both number 141 (73.4%) and expenditure 85.54 (80.13%). This suggests that while most items are not clinically critical, they represent a substantial share of overall costs.

Statistical Interpretation

A Chi-square goodness-of-fit test was applied to examine whether the observed distribution of SKUs across the VED categories differed significantly from an equal expected distribution (Table 3).

**Table 3:** Chi-square goodness-of-fit for VED inventory distribution V = Vital; E: Essential; D: Desirable; O = observed frequencies; E = expected frequencies based on uniform distribution; df = degrees of freedom. Observed frequencies significantly deviate from expected equal distribution across VED categories (χ² = 155.56, df = 2, p < 0.001).

VED category	Observed SKUs (O)	Expected SKUs (E)	% of SKUs	Total cost (₹M)	% of cost	Average cost/SKU (₹M)	(O−E)²/E
Vital (V)	16	64	8.3%	6.46	6.05%	0.40	36.00
Essential (E)	35	64	18.2%	14.75	13.82%	0.42	13.14
Desirable (D)	141	64	73.4%	85.54	80.13%	0.61	106.42
Total	192	—	100%	106.75	100%	—	155.56

The test showed a highly significant deviation from uniformity, χ²(2, N = 192) = 155.56, p < 0.001. The Desirable (D) category contained a disproportionately higher number of SKUs compared with Vital (V) and Essential (E) items, indicating a skewed inventory structure. These results suggest that consumption patterns and procurement decisions strongly favor D-category items, which may not align with clinical criticality. 

Integrated ABC-VED matrix

The combined ABC-VED matrix analysis distributed items across both cost and criticality classifications. The intersection of ABC and VED categories showed a single item 1 (3.7%) in AV, 0 in AE, and 4 items (36.3%) in AD combinations. The B category had 0 items in BV, 7 (10.7%) in BE, and 13 (29%) in BD. The C category contained 15 items (2.4%) in CV, 28 (3.2%) in CE, and 124 (14.9%) in CD combinations (Table 4).

**Table 4:** Integrated ABC-VED matrix of inventory items ABC = Always, Better, Control; VED = Vital, Essential, Desirable Matrix represents cross-classification of drugs based on cost (ABC) and clinical importance (VED). Each cell denotes the number and percentage of total SKUs (N = 192). Column headers show VED cost share; row headers show ABC cost share.

Inventory Items	Vital (6.05%)	Essential (13.82%)	Desirable (80.13%)
Always (A) (39.97%)	1 item (3.7%)	0 items (0%)	4 items (36.3%)
Better (B) (39.64%)	0 items (0%)	7 items (10.7%)	13 items (29%)
Control (C) (20.39%)	15 items (2.4%)	28 items (3.2%)	124 items (14.9%)

Integrated ABC-VED Matrix Distribution

**Figure 2:**
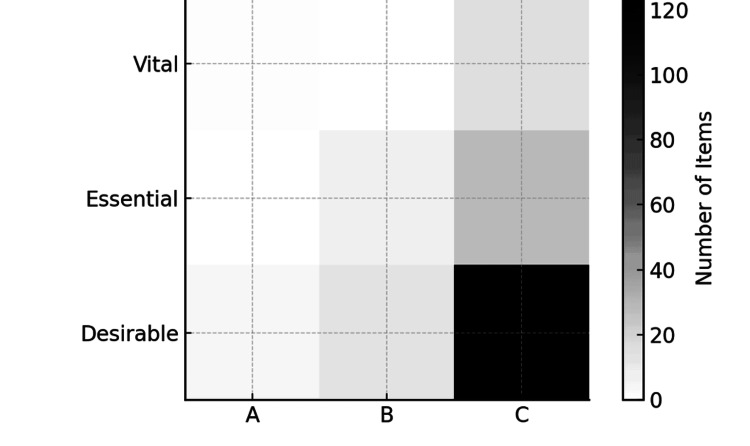
Integrated ABC-VED matrix distribution A: Always; B: Better; C: Control, Vital; E: Essential; D: Desirable; SKUs = stock-keeping units

The integrated ABC-VED matrix shows the intersection between cost-based (ABC) and criticality-based (VED) classifications (Figure 2). High-value, high-priority items (Category AV) form the most critical segment requiring strict monitoring and uninterrupted supply. In contrast, low-cost and low-priority groups (CD, CE) allow flexible inventory control, aiding optimization of procurement and storage strategies.

Statistical Interpretation Integrated ABC-VED Combined Matrix of Inventory Items

A Chi-square test of independence was performed to examine the association between ABC and VED classifications. The test demonstrated a statistically significant relationship between the two systems, indicating that cost-criticality (ABC) and clinical-criticality (VED) were not independent, χ² (df = 4, N = 192) = significant, p < 0.001. A-category items (high-cost) were disproportionately concentrated within the Vital and Essential groups, whereas C-category items (low-cost) overwhelmingly clustered in the Desirable category. This suggests that clinically important drugs are more likely to fall within higher cost brackets, while less critical items dominate the low-cost C category. The combined matrix, therefore, highlights specific inventory zones-particularly the AV, AE, and BE segments-where tighter monitoring and rational procurement policies are required (Table 5).

**Table 5:** Observed vs. expected distribution (ABC vs. VED) with Chi-square evaluation ABC = Always, Better, Control; VED = Vital, Essential, Desirable Expected frequencies were calculated by multiplying total items in each ABC group by the overall VED proportions (Vital 6.05%, Essential 13.82%, Desirable 80.13%). Percentage values in parentheses represent the proportion of items within the ABC category. Discrepancies between observed and expected frequencies contributed to the Chi-square statistic.

ABC category	Observed Vital n (%)	Expected Vital (6.05%)	Observed Essential n (%)	Expected Essential (13.82%)	Observed Desirable n (%)	Expected Desirable (80.13%)
Always (A) (n=5)	1 (3.7%)	0.30	0 (0%)	0.69	4 (36.3%)	4.01
Better (B) (n=20)	0 (0%)	1.21	7 (10.7%)	2.76	13 (29%)	16.02
Control (C) (n=167)	15 (2.4%)	10.10	28 (3.2%)	23.06	124 (14.9%)	133.82
Total (192)	16	—	35	—	141	—

Operational findings

Interviews with pharmacy staff and on-site observations identified several operational challenges. Stock management processes were primarily manual, with paper-based record Binders and delayed data entry. Reordering was reactive, leading to frequent shortages of vital drugs and accumulation of low-priority stock. There was limited forecasting, no automated reordering mechanism, and minimal staff training in data-driven inventory practices.

## Discussion

This study evaluated the pharmaceutical inventory of a tertiary care teaching hospital in Jharkhand through ABC, VED, and integrated ABC-VED analyses. Together, these methods offered a comprehensive view of both financial prioritization and clinical importance, highlighting opportunities to strengthen stock management within a resource-limited healthcare environment.

The ABC analysis revealed that a small fraction of high-cost drugs (2.6% of SKUs) accounted for nearly 40% of total annual expenditure. This pattern of skewed cost distribution is consistent with the Pareto principle, as observed in similar hospital-based studies across India and other developing nations, where 10-20% of items often consume 70-80% of total drug budgets [11-13]. Such concentration of expenditure indicates the need for rigorous monitoring, strict procurement control, and continuous availability of these vital high-cost drugs to prevent financial wastage and clinical stockouts.

The VED classification showed that the majority of stocked items (73.4%) were categorized as Desirable, consuming 80.1% of expenditure, while Vital items represented only 8.3% of SKUs and 6.05% of costs. This imbalance suggests possible overstocking of non-critical drugs and under-prioritization of essential or life-saving medicines. Comparable findings were reported by Wandalkar et al. (2013) and Mahatme et al. (2012), emphasizing that rationalizing desirable items can significantly reduce avoidable costs without affecting patient outcomes [14,15].

The integrated ABC-VED matrix provided a more refined stratification of inventory control priorities. Only a limited subset of items fell into the Category I (AV, AE, AD, BV) group-high-cost and clinically critical drugs requiring stringent oversight. Conversely, a large portion of items belonged to Category III (CD), denoting low-cost, low-priority drugs where procurement flexibility is acceptable. These results align with the observations by Quick et al. (1997) and Sikdar et al. (1996), who demonstrated that integrated analysis allows rational resource allocation, minimizing the risk of stockouts for essential medicines while avoiding overinvestment in low-impact drugs [16,17].

Operational challenges, including manual stock-keeping, lack of digital tracking, and reactive replenishment practices, were consistent with findings from similar low-resource settings [15,16]. Incorporating digital solutions such as barcoding or RFID systems can improve accuracy, enable real-time monitoring, and support automated reorder triggers [17]. Furthermore, routine ABC-VED audits and staff training will foster proactive inventory management.

The comparative findings in Table 6 demonstrate notable variation in ABC, VED, and ABC-VED matrix distributions between the present study and earlier Indian analyses. The proportion of Category A drugs (2.6%) in this study is substantially lower, while Category C drugs (86.9%) are markedly higher than those reported by Vaz et al. (2008), Devnani et al. (2010), Gupta et al. (2007), Sikdar et al. (1996), and Thawani et al. (2004) [2-4,17,18]. Similarly, the VED classification shows reduced proportions of Vital (8.3%) and Essential (18.2%) items, alongside an unusually high share of Desirable drugs (73.4%), indicating a formulary composition that differs considerably from the more balanced patterns observed in previous studies. The ABC-VED matrix also reflects lower Category I (15.4%) and Category III (14.9%) items compared with earlier reports. Collectively, these differences suggest that the present hospital’s inventory is dominated by low-cost and non-critical medicines, highlighting the need for strengthened, priority-based procurement and improved alignment with essential therapeutic requirements.

**Table 6:** Comparision of ABC, VED and ABC-VED matrix analysis of different studies on India A: Always, B: Better, C: Control, V: Vitale, Essential, D: Desirable, I: AV+AE+AD+BV+CV, II: BE+CE+BD, III: CD All figures are in %.

Category	Present study	Devnani et al. (2010) [3]	Vaz et al. (2008) [2]	Gupta et al. (2007) [4]	Thawani et al. (2004) [18]	Sikdar et al. (1996) [17]
A	2.6	13.78	12.93	14.46	10.76	17.81
B	10.4	21.85	19.54	22.46	20.63	22.60
C	86.9	64.37	67.53	63.08	68.61	59.59
V	8.3	12.11	12.36	7.39	23.76	5.14
E	18.2	59.38	47.12	49.23	38.12	58.90
D	73.4	28.51	40.52	43.38	38.12	35.96
I	15.4	22.09	22.99	20.92	29.15	21.58
II	42.94	54.63	41.67	48.92	41.26	56.16
III	14.9	23.28	35.34	30.16	29.59	22.26

Limitations

Limitations of this study include its single-center design and exclusion of non-pharmaceutical medical supplies. Future research could evaluate the impact of digital interventions on inventory efficiency and clinical outcomes.

Recommendations

Based on the findings of this study, several steps can help improve pharmaceutical inventory management in tertiary care hospitals. Digitizing inventory systems with barcode or RFID tracking-especially for high-value and vital medicines-should be a priority to improve accuracy and transparency. Conducting ABC-VED audits every quarter will help track changes in medicine use and spending, allowing timely corrections. Strengthening the Drug and Therapeutics Committee (DTC) can support more evidence-based purchasing and better coordination between departments. Regular training for pharmacy staff in ABC-VED analysis, forecasting, and digital record management will further improve daily operations. Using additional decision-making tools, such as Pareto-based methods, can help refine priorities and improve resource allocation. Finally, aligning procurement practices with WHO and national essential medicine guidelines will promote rational use of medicines, sustainability, and better patient care.

## Conclusions

The integration of ABC and VED techniques proved to be a practical and evidence-based approach for rationalizing pharmaceutical inventory in a tertiary care teaching hospital. By linking financial significance with clinical necessity, these tools enable informed decision-making, improved stock visibility, and reduced wastage. The study demonstrates that routine ABC-VED monitoring, supported by digital systems, can optimize medicine availability and efficiency-enhancing the resilience of hospital pharmacy services in resource-limited settings.

## References

[1] Chen S (2025). Global Spending on Health: A World in Transition. J Appl Pharm..

[2] Vaz FS, Ferreira AM, Kulkarni MS, Motghare DD, Pereira-Antao I (2008). A study of drug expenditure at a tertiary care hospital: an ABC-VED analysis. J Health Manag.

[3] Devnani M, Gupta A, Nigah R (2010). ABC and VED analysis of the pharmacy store of a tertiary care teaching, research and referral healthcare institute of India. J Young Pharm.

[4] Gupta R, Gupta KK, Jain BR, Garg RK (2007). ABC and VED analysis in medical stores inventory control. Med J Armed Forces India.

[5] Jobira T, Abuye H, Jemal A, Gudeta T (2025). Evaluation of pharmaceuticals inventory management in selected health facilities of West Arsi Zone, Oromia, Ethiopia. Integr Pharm Res Pract.

[6] Yohannes T, Boche B, Birhanu N, Gudeta T (2022). Matrix analyses of pharmaceutical products for the years 2017 to 2019 among public health facilities in Hadiya zone, Ethiopia: a cross-sectional descriptive study. BMC Health Serv Res.

[7] Meena DK, Mathaiyan J (2025). Assessment of drug inventory using ABC-VED matrix analysis in selected public health facilities of Puducherry, India. J Family Med Prim Care.

[8] Mulac A, Mathiesen L, Taxis K, Gerd Granås A (2021). Barcode medication administration technology use in hospital practice: a mixed-methods observational study of policy deviations. BMJ Qual Saf.

[9] Zheng WY, Lichtner V, Van Dort BA, Baysari MT (2021). The impact of introducing automated dispensing cabinets, barcode medication administration, and closed-loop electronic medication management systems on work processes and safety of controlled medications in hospitals: A systematic review. Res Social Adm Pharm.

[10] Lee I, Lee BC (2010). An investment evaluation of supply chain RFID technologies: a normative modeling approach. Int J Prod Econ.

[11] Álvarez López Y, Franssen J, Álvarez Narciandi G, Pagnozzi J, González-Pinto Arrillaga I, Las-Heras Andrés F (2018). RFID technology for management and tracking: e-health applications. Sensors (Basel).

[12] Dora S, Singh AK, Panda PS, Sahoo JS (2020). Inventory management of drugs at a secondary level health-care centre in Odisha. Indian J Community Fam Med.

[13] Shaikh Teli E, Bhangale C, Momin K, Ramanand J, Mahajan H (2022). Application of ABC-VED analysis for inventory control in drug store of a tertiary care hospital of North Maharashtra. Perspect Med Res.

[14] Wandalkar P, Pandit PT, Zite AR (2013). ABC and VED analysis of the drug store of a tertiary care teaching hospital. Indian J Basic Appl Med Res.

[15] Mahatme M, Dakhale G, Hiware S, Shinde A, Salve A (2012). Medical store management: an integrated economic analysis of a tertiary care hospital in central India. J Young Pharm.

[16] Quick JD, Hogerzeil HV, Rankin JR, Dukes MN, Laing R, Garnett A, O’Connor RW (1997). Managing Drug Supply: The Selection, Procurement, Distribution, and Use of Pharmaceuticals (2nd Ed.). West Hartford, Connecticut : Kumarian Press.

[17] Sikdar SK, Agarwal AK, Das JK (1996). Inventory analysis by ABC and VED analysis in medical stores depot of CGHS, New Delhi. Health Popul Perspect Iss.

[18] Thawani VR, Turankar AV, Sontakke SD, Dakhale GN, Jaiswal KS, Gharpure KJ, Dharmadhikari SD (2004). Economic analysis of drug expenditure in Government Medical College Hospital, Nagpur. Indian J Pharmacol.

